# TSI-GNN: Extending Graph Neural Networks to Handle Missing Data in Temporal Settings

**DOI:** 10.3389/fdata.2021.693869

**Published:** 2021-09-15

**Authors:** David Gordon, Panayiotis Petousis, Henry Zheng, Davina Zamanzadeh, Alex A.T. Bui

**Affiliations:** ^1^ Department of Bioengineering, University of California Los Angeles, Los Angeles, CA, United States; ^2^ Medical and Imaging Informatics (MII) Group, Department of Radiological Sciences, University of California Los Angeles, Los Angeles, CA, United States; ^3^ UCLA Clinical and Translational Science Institute, Los Angeles, CA, United States

**Keywords:** missing data, imputation, temporal data, irregular sampling, deep learning, graph neural networks

## Abstract

We present a novel approach for imputing missing data that incorporates temporal information into bipartite graphs through an extension of graph representation learning. Missing data is abundant in several domains, particularly when observations are made over time. Most imputation methods make strong assumptions about the distribution of the data. While novel methods may relax some assumptions, they may not consider temporality. Moreover, when such methods are extended to handle time, they may not generalize without retraining. We propose using a joint bipartite graph approach to incorporate temporal sequence information. Specifically, the observation nodes and edges with temporal information are used in message passing to learn node and edge embeddings and to inform the imputation task. Our proposed method, temporal setting imputation using graph neural networks (TSI-GNN), captures sequence information that can then be used within an aggregation function of a graph neural network. To the best of our knowledge, this is the first effort to use a joint bipartite graph approach that captures sequence information to handle missing data. We use several benchmark datasets to test the performance of our method against a variety of conditions, comparing to both classic and contemporary methods. We further provide insight to manage the size of the generated TSI-GNN model. Through our analysis we show that incorporating temporal information into a bipartite graph improves the representation at the 30% and 60% missing rate, specifically when using a nonlinear model for downstream prediction tasks in regularly sampled datasets and is competitive with existing temporal methods under different scenarios.

## Introduction

Graph representation learning (GRL) aims to accurately encode structural information about graph-based data into lower-dimensional vector representations (Hamilton, 2020). The basic idea is to encode nodes into a latent embedding space using geometric relationships that can then be used to accurately reconstruct the original representation (Hoff et al., 2002). There are two node embedding approaches: shallow embedding methods and more complex encoder-based models (i.e., graph neural networks, GNNs) (Hamilton, 2020). Shallow embedding methods, such as inner product and random walks, are inherently transductive meaning they can only generate embeddings for nodes present during training, which can restrict generalizability without retraining (Ahmed et al., 2013; Perozzi et al., 2014; Grover and Leskovec, 2016; Hamilton, 2020). In contrast, GNNs use more complex encoders that depend more on the structure and attributes of the graph, allowing them to be used on inductive applications (i.e., evolving graphs) (Hamilton et al., 2017a; Hamilton et al., 2017b). A key feature of GNNs is that they can use *k*-rounds of message passing (inspired by belief propagation), where messages are aggregated from neighborhoods and then combined with the representation from the previous layer/iteration to provide an updated representation (Hamilton, 2020).

Recently, GRAPE (You et al., 2020), a framework for handling missing data using graph representation, proposed formulating the problem using a bipartite graph, where the observations and features in a data matrix comprise two types of nodes, observation and feature nodes, and the observed feature values are the attributed edges between the two types of nodes. GRAPE used a modified GraphSAGE (Hamilton et al., 2017b) architecture and introduced edge embeddings during message passing to learn edge attributes and was shown to outperform a deep generative model (Yoon et al., 2018a), as well as traditional methods on edge-level prediction and node-level prediction tasks (You et al., 2020). Yet one of the shortcomings of GRAPE is it assumes observations are independent, which is generally not the case in temporal settings with repeated measurements. Therefore, representations learned using GRAPE may not be suitable for temporal data with repeated measurements.

There are numerous contemporary imputation methods. Recurrent neural networks (RNNs) capture sequence information well when handling missing data (Lipton et al., 2016; Che et al., 2018), particularly bidirectional RNNs that use information from the past, present, and future (via forward and backward connections) (Yoon et al., 2018b; Cao et al., 2018), but RNNs learn a chain structure, whereas GNNs learn across geometric spaces via message passing in a graph-structured manner. Non-autoregressive models have been proposed to capture long-range sequence information in parallel, which rely on bidirectional RNNs to process input data, but the implementation does not handle irregular sampling (Liu et al., 2019). GNNs have been combined with matrix completion to extract spatial features, but these approaches do not explicitly capture temporal information and the implementations only use discrete datatypes (e.g., ratings) (Berg et al., 2017; Monti et al., 2017; Zhang and Chen, 2019). Further, separable recurrent multi-graph convolutional neural networks (sRMGCNN), which feed the extracted spatial features from a MGCNN into an RNN to exploit the temporal information, are transductive (Monti et al., 2017). Autoencoders (AEs) can efficiently learn undercomplete (i.e., lower-dimensional) or overcomplete (i.e., higher-dimensional) representations (Beaulieu-Jones et al., 2017; Gondara and Wang, 2018; McCoy et al., 2018), but the recovered values are not based on an aggregation from a non-fixed number of neighbors, as in GNNs. Further, AEs cannot explicitly train over incomplete data (i.e., AEs initialize with arbitrary/default values) (Gondara and Wang, 2018) or explicitly exploit temporal information (i.e., AEs combine with a temporal dynamic model, such as a Gaussian process (Fortuin et al., 2020) or RNN (Park et al., 2020)).

Similarly, there are a myriad of classic imputation methods. Matrix completion can exploit correlations within and across feature dimensions, but it is generally only used in a static setting (i.e., single measurement that does not change over time) (Candès and Recht, 2009). Interpolation methods have been proposed to exploit correlations within feature dimensions in temporal settings; however, they ignore correlations across feature dimensions (Kreindler and Lumsden, 2012). K-nearest neighbors (KNN) learns an aggregation, but from a fixed number of neighbors with weights based on Euclidean distance and is usually only applied to static data (Troyanskaya et al., 2001). MissForest is a non-parametric method that uses a random forest trained on the observed values of a dataset to predict the missing values, but is generally a static method (Stekhoven and Bühlmann, 2012).

In contrast to single imputation, multiple imputation methods aim to model the inherent variability into recovered values to account for the uncertainty in estimating missing values (Yoon, 2020). While multiple imputation by chained equations (MICE) (White et al., 2011) is the gold standard, it is generally a static method and may not perform well at higher rates of missingness (Yoon, 2020). Some contemporary methods also produce multiple imputations, such as RNN-based and GNN-based methods that utilize a dropout hyperparameter (Srivastava et al., 2014; Yoon et al., 2018b; Rong et al., 2019; You et al., 2020) as well as AE-based methods that initialize with different sets of random weights at each run (Gondara and Wang, 2018).

In this work, we introduce temporal setting imputation using graph neural networks (TSI-GNN), which extends graph representation learning to handle missing data in temporal settings. We build on previous GRL methods by capturing sequence information within the same type of nodes (i.e., observation nodes) in a bipartite graph and by exploring how we can recover an accurate temporal representation that preserves the original representations’ feature-label relationships. TSI-GNN incorporates temporal information into a bipartite graph without creating actual edges between the same type of nodes, enhancing the learned representation without violating bipartite graph properties. While we evaluate TSI-GNN using the modified GraphSAGE architecture from GRAPE (You et al., 2020), our approach is general to GNN-based approaches that use a bipartite graph representation.

## Methods

### Representation and Observation Node and Edge Definition

An ideal imputation method learns to recover the original relationships in a dataset (Yoon, 2020). Extending a graph representation to the temporal setting should therefore preserve temporal dynamics such that the recovered representation keeps the original relationships between the feature and label across time (Meng, 1994). In temporal settings with repeated measurements, observations are often correlated, particularly frequent measurements (e.g., stocks, energy, healthcare) (Yoon, 2020). Therefore, an imputation method for temporal settings with repeated measurements should capture temporal information, not ignore it. Similarly, features in temporal settings can be correlated. Thus, a GNN-based temporal imputation method should learn to recover important information within and between sets of the two types of nodes (i.e., observation nodes and feature nodes). We illustrate this with the following scenario in the healthcare setting:

Patient 1 labs (e.g., estimated glomerular filtration rate and potassium) and vitals (e.g., respiratory rate and systolic blood pressure) are monitored every 4 h for a sequence length of 3 checks (i.e., 12 h total). In this scenario, Patient 1 had three observations (repeated measurements).

According to You et al., the key innovation of GRAPE is to formulate the problem using a bipartite graph representation (You et al., 2020). In a bipartite graph, the absence of an edge between the same type of nodes [e.g., observation node 1 (*O*_1_) and observation node 2 (*O*_2_)] implies that *O*_1_ and *O*_2_ are independent, denoted by *O*_1_ ⫫ *O*_2_. While this may hold in a static setting, in the case of temporal settings with repeated measurements (as illustrated in the healthcare scenario above), this does not necessarily hold and to assume it does could ignore important temporal information. To adhere to bipartite graph properties, we do not create edges between the observation nodes to exploit temporal information. Instead, we incorporate sequence length, which represents a sequence among sequences of datapoints, into the observation nodes and edges, thereby capturing temporal information in observation nodes and edges that may provide a more accurate chronological representation of the data. We call this type of approach a joint bipartite graph, as it incorporates sequence length, which addresses the independence assumption between observation nodes implied in a bipartite graph.

### Intuition Behind Joint Bipartite Graph in TSI-GNN

**FIGURE 1:**
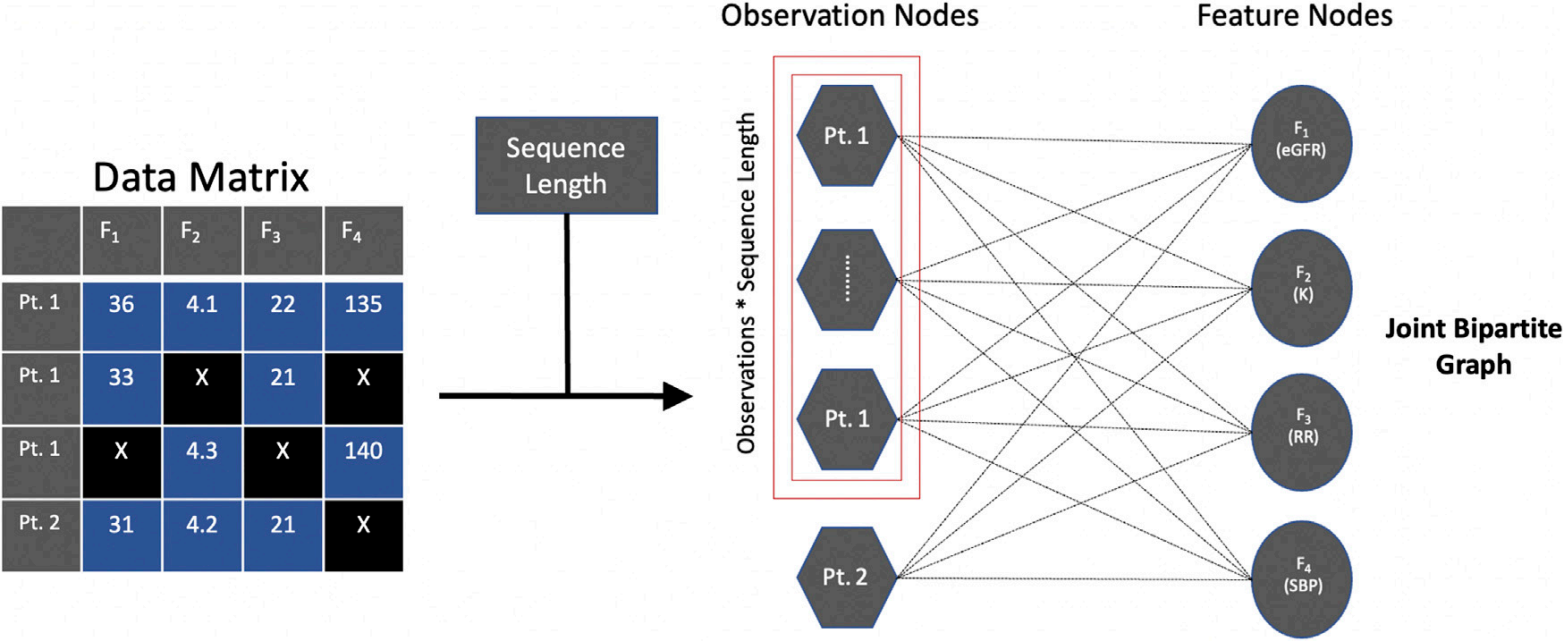
Joint bipartite graph. Captures temporal information [e.g., observation nodes for patient 1 (Pt. 1)] without creating actual edges as well as captures information between the observation and feature nodes (i.e., edge attributes). Abbreviations: estimated glomerular filtration rate (eGFR), potassium (K), respiratory rate (RR), systolic blood pressure (SBP), patient 2 (Pt. 2).

Our key innovation is to formulate the problem using a joint bipartite graph (Figure 1). Let *G (V, E)* be the joint bipartite graph of nodes *V* and undirected edges *E*. *V* is comprised of two types of nodes, *V*_*observations*_ *∪ V*_*features*_ *,* such that *V*_*observations*_ *= {u*_*1*_ *, … ,u*_*m*_ *},* where the size of *m* is the number of observations minus the sequence length times the sequence length, and *V*_*features*_ *= {v*_*1*_ *, … ,v*_*n*_ *}*, where the size of *n* is the number of features. *E* contains the undirected edges between *V*_*observations*_ and *V*_*features*_, where *E = {k*_*1*_ *, … ,k*_*p*_ *}* and the size of *p* is the number of observations minus the sequence length times the sequence length times the number of features.

To incorporate sequence length into observation nodes and edges, we use an operation similar to the idea of reshaping a 3D array with a sequence length dimension to a 2D array (The NumPy community, 2021), which keeps the sequence length information and can then be used as input for a GNN to exploit. Prior to reshaping, we cut the data by the sequence length (i.e., apply a sliding window technique), which is an operation also implemented in existing temporal imputation methods (Yoon et al., 2018b; Yoon et al., 2019). For example, using the stock dataset (see *Datasets*), after cutting the data by the sequence length, let observations, sequence length, and features, respectively denote the parameters in the 3D array, (4120-21 = 4099, 21, 6). After reshaping the 3D array, let observations and features denote the parameters in the 2D array, (4099*21 = 86079, 6), which keeps the sequence length information (i.e., sequence length of 21), but in a different shape. Since we know the sequence length, we can verify that it is kept by the 2D representation by demonstrating that we can recover the 3D array with dimensions (4099, 21, 6) by reshaping the 2D array with dimensions (86079, 6). In a similar vein, RNN-based and GAN-based methods have empirically shown that the operation of reshaping 3D arrays with a sequence length dimension into 2D arrays is a suitable method for keeping sequence length information (Yoon et al., 2018b; van der Schaar Lab: T-I, 2020). Specifically, M-RNN uses this reshaping operation in the training and predicting fully connected network functions (Yoon et al., 2018b) and T-GAIN uses this reshaping operation in the fit and transform functions (van der Schaar Lab: T-I, 2020). While our application of the reshaping operation is for graph representation, the logic remains the same.

Thus, this joint bipartite graph captures temporal information across the same type of nodes (i.e., observation nodes) without creating actual edges (which we informally refer to as using “ghost” edges) as well as captures information between the observation and feature nodes (i.e., the edge attributes) for GNN-based approaches to leverage.

### Optimizing the Number of Trainable Edges in TSI-GNN

Incorporating sequence length can significantly increase the number of trainable edges in a GNN. As such, it is helpful to be aware of the size of the potential TSI-GNN before its training. Let observations, sequence length, features, respectively denote the main parameters affecting the size of the TSI-GNN,

$$\begin{array}{c} Size\;\approx \;\left(Observations-\;Sequence\;Length\right)\;x\;Sequence\;Length\;x\;Features\leq Threshold \end{array}$$ (1)

where a nonnegative threshold is a hyperparameter used to balance the selection of parameters affecting size and size ≤threshold is practical to implement for the machine capable of running the model. For example, on a MacBook M1 with 8-core CPU and 16 GB RAM, generating a TSI-GNN with a size ≤ four million is practical for implementation. Furthermore, at lower rates of missingness there are a larger number of trainable edges (i.e., it produces a larger TSI-GNN) relative to higher rates of missingness where there are a lower number of trainable edges (i.e., resulting in a smaller TSI-GNN).

As GNNs are inductive, it is feasible to train on smaller subsets of the larger dataset, learn a temporal representation, then generalize to unseen data; thereby, reducing the computational complexity of the model the data is trained on.

### Baseline Imputation Methods

**FIGURE 2:**
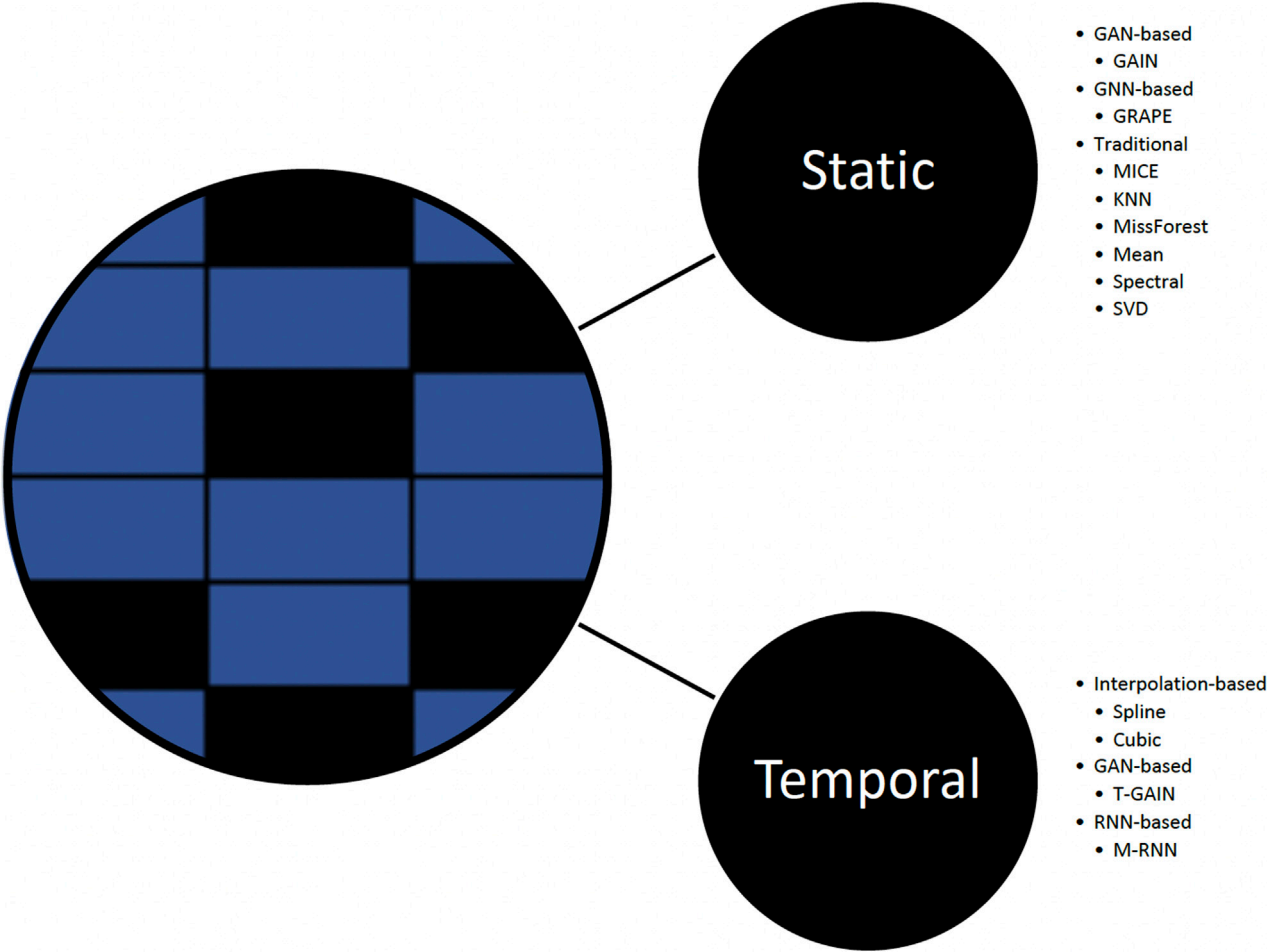
Missing data imputation methods.

In this work, we explore the performance of baseline imputation methods (Figure 2) that include well-established and contemporary approaches commonly used in static and temporal settings:

1) Static methods. Generative adversarial imputation networks (GAIN), introduces a hint mechanism to ensure that the generator generates samples according to the true underlying data distribution (Yoon et al., 2018a); GRAPE, formulates the problem using a bipartite graph, modifies the GraphSAGE architecture, and introduces edge embeddings (You et al., 2020); MICE, consists of three steps: impute *m* times from a distribution, analyze each of the *m* datasets resulting in *m* analyses, and pool *m* results into one result; KNN finds similar samples and performs imputation using a weighted average of the neighbors; missForest; mean; spectral; and singular value decomposition (SVD).
2) Temporal methods. T-GAIN, GAIN extension for the temporal setting with implementation from Clairvoyance (Yoon et al., 2018a; van der Schaar Lab: T-I, 2020; Jarrett et al., 2020); M-RNN, uses bi-directional RNN and performs interpolation simultaneously with feature imputation (Yoon et al., 2018b); spline interpolation; and cubic interpolation.

### Datasets

We utilized publicly available datasets from three domains: finance, energy, and healthcare. Table 1 outlines the dataset profiles:

1) Stocks. The daily historical Google stock data from August 2004 through December 2020. This dataset consists of six features (opening, high, low, closing, adjusted closing prices, and volume).
2) Energy. We used a subset of 3,001 observations from the original 19,735 observations in the UCI Appliances energy prediction dataset. This dataset consists of 28 features (temperature, humidity, weather, and usage related attributes).
3) Healthcare: ICU Setting. We used a completely observed subset of patients from the Medical Information Mart for Intensive Care-III (MIMIC-III) database (Johnson et al., 2016), of individuals who received antibiotics at any point, based on the daily decision on antibiotic treatment. This dataset was extracted based on the preprocessing guidelines from the Clairvoyance implementation but filtered to produce a complete dataset (Jarrett et al., 2020). We selected 16 features (common labs and vital signs) from the original 27 features. Further, we randomly sampled 550 patients from the subset with a minimum sequence length of 9. While using a lower number of patients may degrade performance of leading benchmark temporal methods, such as M-RNN (Yoon et al., 2018b), it enables testing our method at higher sequence lengths, which we believe is suitable for this work.

**TABLE 1:** Dataset profiles.

	Stocks	Energy	ICU
# of observations	4,120 (2004-2020)	3,001	8,250 (550 patients)
# of features (cont, cat)	6 (6,0)	28 (28,0)	16 (15,1)
Label	Volume	Light Usage	Ventilator
Missing rate (MR)	30%	30%	30%
	60%	60%	60%
Measurement frequency	24 h	Every 10 min	4 h
Sequence length	21	24	15^a^
# of trainable edges			
30% MR	602K	2.7M	2.6M
60% MR	345K	1.5M	1.5M

a Average sampling frequency.

### Determining Sequence Length

Sequence length is a vital parameter for temporal imputation methods and should be thoughtfully determined. In this work, the regularly sampled datasets (i.e., stocks, energy) contain the same number of repeated measurements as the number of observations; therefore, selecting a sequence length for these datasets is somewhat flexible. For example, previous methods using a similar stock dataset set the sequence length ranging from 7 to 24 days (Yoon et al., 2018b; Yoon et al., 2019). In this work, we set the sequence length at 21 days.

In this work, the irregularly sampled dataset (i.e., healthcare) contains multiple observations per patient, and the number of observations vary between patients. Therefore, determining an appropriate sequence length requires careful consideration. Previous research has suggested calculating an average sequence length for electronic health record (EHR) datasets and found that an average sequence length above 10 has been shown to lead to improved performance in contrast to lower average sequence lengths (Yoon et al., 2018b). Further, RNN-based and GAN-based methods have employed a maximum on the sequence length in EHR datasets to handle irregular sampling (Jarrett et al., 2020). After applying the inclusion criteria, the healthcare dataset in this work has an average sequence length of 15. To handle the irregular sampling, we set the maximum sequence length to be the same as the average sequence length, 15.

### Model Training and Evaluation

The datasets are fully observed; therefore, we mask 30% and 60% of the data completely at random, recreating the missingness scenario where data are missing completely at random. Since the majority of the variables in the datasets we explore are continuous, we evaluate imputation performance using the root mean square error (RMSE).

To test the effect of imputation on the downstream prediction task, we follow a holdout procedure via a 70:30 training and test set split. In this study, data was normalized before input to the models, and we did not renormalize or round the output of the models. Therefore, we evaluate prediction performance using R^2^, which is a measure to assess the goodness of fit. We compare the R^2^ of the imputed values to the original values to assess the congeniality of the models – that is how well the imputed values preserve the feature-label relationships of the original dataset (Meng, 1994). We use a nonlinear model, gradient boosting regression trees (GBR), as well as a linear model, linear regression (LR), to determine which model may be more appropriate for the dataset as well as more congenial to the original representation.

### Configurations

1) TSI-GNN uses the same modified GraphSAGE architecture as GRAPE (You et al., 2020) to fairly evaluate the performance of a joint bipartite graph representation (TSI-GNN) to a bipartite graph representation (GRAPE). Further, for TSI-GNN and GRAPE, we set the number of epochs to 2,000 and use the Adam optimizer with a learning rate at 0.001. We use a 3-layer GNN with 64 hidden units (You et al., 2020). We use the mean aggregation function and ReLU activation function. For TSI-GNN, we set the sequence length at 21, 24, and 15 for the stocks, energy, and ICU datasets, respectively.
2) For M-RNN, we use four hidden state dimensions and a batch size of 64. We train the model for 2,000 iterations with a learning rate at 0.001. We set the sequence length at 21, 24, and 15 for the stocks, energy, and ICU datasets, respectively.
3) For GAIN and T-GAIN, we train the model for 2,000 iterations with a learning rate at 0.001. We use a batch size of 64, a hint rate of 0.9, and an alpha of 100. For T-GAIN, we set the sequence length at 21, 24, and 15 for the stocks, energy, and ICU datasets, respectively.

### Implementation

To perform the analysis, we use Python v3.7.9. We also use python packages Pandas (team (2021). Tpd. Pandas., 2021), NumPy (Harris et al., 2020), Scikit-learn (Pedregosa et al., 2011), TensorFlow (Abadi et al., 2016), PyTorch (Paszke et al., 2019), PyTorch Geometric (Fey and Lenssen, 2019), missingPy (Bhattarai, 2018), and FancyImpute (Rubinsteyn and Feldman, 2016).

## Results

### TSI-GNN Improvement Over GRAPE in Downstream Prediction

TSI-GNN outperforms GRAPE at the 30% and 60% missing rate with respect to the original dataset in the downstream prediction task, specifically when using GBR in the regularly sampled datasets (Table 2). In the stock dataset, TSI-GNN R^2^ for GBR is 0.141 and 0.123 higher than GRAPE R^2^ at 30% and 60% missing rate, respectively (Table 2). In the energy dataset, TSI-GNN R^2^ for GBR is 0.097 and 0.146 higher than GRAPE R^2^ at 30% and 60% missing rate, respectively (Table 2). In the ICU dataset, TSI-GNN GBR is 0.030 and 0.033 higher than GRAPE R^2^ at 30% and 60% missing rate, respectively (Table 2).

**TABLE 2:** Imputation Methods Prediction Task (GBR/LR). Where a similar R^2^ for the imputed values to the original values suggests a potentially more accurate representation.

Setting	Method	Missing rate	Stocks GBR/LR	Energy GBR/LR	ICU GBR/LR
n/a	**Original**	n/a	0.588/0.33	0.623/0.31	0.657/0.66
**Temporal**	**T-GAIN**	30%	**0.606**/0.36	0.554/0.36	0.732/0.70
		60%	0.662/**0.30**	0.500/0.38	0.840/0.78
	**M-RNN**	30%	0.677/0.30	0.504/**0.28**	0.602/0.57
		60%	**0.636**/0.23	**0.605**/0.45	0.472/0.43
	**Spline**	30%	0.654/**0.34**	0.352/0.24	0.458/0.43
	**Interpolation**	60%	0.754/0.39	0.105/0.09	0.223/0.21
	**Cubic**	30%	0.609/**0.32**	**0.652**/0.37	0.579/0.55
	**Interpolation**	60%	0.511/0.26	0.660/**0.26**	0.362/0.29
	**^a^TSI-GNN**	30%	**0.606**/0.28	0.563/0.27	**0.666/0.64**
		60%	0.537/0.26	0.379/0.18	**0.640/0.63**
**Static**	**GAIN**	30%	**0.578**/0.36	0.372/0.24	0.732/0.73
		60%	**0.629**/0.47	**0.415/0.36**	0.968/0.85
	**MICE**	30%	0.526/0.30	0.489/**0.31**	0.635/0.64
		60%	0.372/0.26	0.230/0.14	0.613/0.61
	**KNN**	30%	0.538/**0.32**	**0.612**/0.34	0.638/**0.65**
		60%	0.430/0.25	0.221/0.15	0.610/**0.63**
	**MissForest**	30%	0.614/0.37	0.716/0.43	0.732/0.71
		60%	0.777/**0.35**	0.824/0.58	0.853/0.76
	**Mean**	30%	0.478/0.26	0.463/0.25	0.634/0.61
		60%	0.334/0.23	0.220/0.12	0.603/0.54
	**Spectral**	30%	0.531/0.29	0.491/0.29	**0.639**/0.62
		60%	0.391/0.23	0.209/0.12	**0.622**/0.49
	**SVD**	30%	0.531/0.31	0.459/0.27	0.636/0.64
		60%	0.414/0.24	0.221/0.15	0.616/0.60
	**GRAPE**	30%	0.465/0.26	0.466/0.27	0.636/**0.65**
		60%	0.414/0.26	0.233/0.15	0.607/**0.63**

a Our method.

Bold values are the best method(s), closest to the original values, per setting, dataset, missing rate, and model (nonlinear vs linear).

### Downstream Prediction Across Static and Temporal Methods

In the stock dataset, at the 30% missing rate, with respect to the original dataset, TSI-GNN, T-GAIN, and GAIN perform similarly and achieve the best for GBR, followed closely by cubic interpolation, while spline interpolation, cubic interpolation, and KNN perform the best for LR (Table 2). At the 60% missing rate, with respect to the original dataset, GAIN performs the best for GBR, followed closely by M-RNN and TSI-GNN, while missForest performs the best for LR (Table 2). In the energy dataset, at the 30% missing rate, with respect to the original dataset, KNN performs the best for GBR, while MICE performs the best for LR (Table 2). Notably, at the 60% missing rate, with respect to the original dataset, M-RNN performs the best for GBR, while cubic interpolation and GAIN perform the best for LR (Table 2). In the ICU dataset, at the 30% missing rate, with respect to the original dataset, TSI-GNN performs the best for GBR, while KNN and GRAPE perform the best for LR (Table 2). At the 60% missing rate, with respect to the original dataset, TSI-GNN performs the best for GBR, while KNN, GRAPE, and TSI-GNN perform the best for LR (Table 2).

### Imputation Performance Across Static and Temporal Methods

In the stock dataset, at the 30% missing rate, MICE performs the best, followed closely by TSI-GNN (Table 3). But at the 60% missing rate, MICE is outperformed by all temporal methods except for T-GAIN (Table 3). In the energy dataset, at the 30% and 60% missing rate, missForest performs the best (Table 3). In the ICU dataset, at the 30% missing rate, MICE performs the best, followed by TSI-GNN and GRAPE (Table 3). Yet at the 60% missing rate, TSI-GNN and GRAPE outperforms MICE (Table 3).

**TABLE 3:** Imputation Methods RMSE. A smaller RMSE is better.

Setting	Method	Stocks		Energy		ICU	
		30%	60%	30%	60%	30%	60%
**Temporal**	**T-GAIN**	0.073	0.107	0.1305	0.2349	0.1045	0.118
	**M-RNN**	0.036	0.041	**0.1003**	**0.1279**	0.1666	0.189
	**Spline Interpolation**	0.043	0.044	0.1684	0.1672	0.2339	0.2346
	**Cubic Interpolation**	0.031	**0.039**	0.1337	0.1493	0.1777	0.244
	^a^ **TSI-GNN**	**0.029**	0.083	0.1364	0.1443	**0.0748**	**0.0762**
**Static**	**GAIN**	0.071	0.106	0.132	0.2328	0.1009	0.1162
	**MICE**	**0.0281**	0.097	0.1075	0.1418	**0.0713**	0.0901
	**KNN**	0.0386	0.131	0.0966	0.1939	0.0826	0.0862
	**MissForest**	0.0329	**0.081**	**0.0620**	**0.1009**	0.0942	0.1078
	**Mean**	0.2299	0.231	0.2076	0.2070	0.2141	0.2139
	**Spectral**	0.0394	0.180	0.1307	0.2331	0.1395	0.3079
	**SVD**	0.0387	0.131	0.1541	0.1779	0.0827	0.1150
	**GRAPE**	0.0337	0.085	0.1463	0.146	0.0768	**0.0748**

a Our method.

Bold values are the best method per setting, dataset, and missing rate.

## Discussion

In this work, we show that formulating the problem using a joint bipartite graph, which incorporates sequence length information into bipartite graphs, can improve the representation at the 30% and 60% missing rate, specifically when using GBR for downstream prediction tasks in regularly sampled datasets. Moreover, we demonstrate that TSI-GNN is able to capture the temporal information between observation nodes without creating actual edges between them. In contrast, GRAPE formulates the problem using a bipartite graph, which does not incorporate sequence length or capture the temporal relationships between observation nodes. Our proposed method has the potential to capture meaningful temporal dynamics that can be useful in various domains and applications. While determining the sequence length of a dataset can be straightforward in regularly sampled datasets it requires more consideration in irregularly sampled datasets. In this work, we highlight learning the average sequence length in EHR data and incorporating it into bipartite graphs; however, this can be generalized to various irregularly sampled data.

A limitation to our proposed method is that it increases the number of trainable edges in a GNN. But as demonstrated, it can improve the representational capacity. Therefore, using the guidelines we provided regarding managing the size of the generated TSI-GNN, one can practically implement and potentially scale this method. Interestingly, for datasets with higher rates of missingness, this limitation is potentially nullified as the number of trainable edges is lower. Another limitation is the preprocessed ICU dataset we used for testing our method. It is possible that some of the preprocessing steps used (e.g., using a completely observed subset or a fixed sequence length) removed important temporal information that degraded the performance of the temporal methods in the healthcare domain. Further, in the ICU dataset at the 30% missing rate, while the TSI-GNN R^2^ for GBR was most similar to the original R^2^, it was slightly higher (0.009). Similarly, in the stocks dataset at the 30% missing rate, TSI-GNN R^2^ for GBR was slightly higher than the original R^2^ (0.018).

In this work, we empirically show that joint bipartite graph representation captures temporal information; however, future work is needed to provide theoretical foundations that can elucidate how GNNs exploit temporal information. Furthermore, using RMSE may be a more biased performance metric when handling missing data for categorical variables (Wang et al., 2021). Although the vast majority of the variables in the datasets we explored are continuous, there still exists some ambiguity regarding choosing a single appropriate metric to use when evaluating imputation performance on a dataset with a mixture of categorical and continuous variables.

While the main contribution of this work was to introduce TSI-GNN, we also demonstrate the performance of a non-exhaustive collection of benchmark temporal and static imputation methods. Not surprisingly, we did not find a single temporal method that worked the best across all domains. Rather, our findings suggest that each data domain has unique characteristics that can make optimizing various classic and contemporary methods across multiple domains difficult. Recently, a Bayesian optimization/ensemble approach was applied on-top of various imputation methods, which seems to help reduce challenges associated with selecting and tuning the appropriate imputation method for a given domain (Jarrett et al., 2020). Still, this suggests that the choice of an imputation technique must be carefully considered in light of the underlying data distribution as well as downstream application in data analysis – no singular method will be superior without sufficient context regarding its usage.

In future work, we plan to explore temporal imputation boosting with interpolation layers (TIBIL) for healthcare datasets with less frequent measurements (e.g., annual intervals) and shorter sequence lengths (e.g., 4). More specifically, TIBIL uses: 1) an upsample interpolation layer to produce more frequent and longer sequence lengths; 2) temporal imputation, such as TSI-GNN, to handle missing data; and 3) a downsample interpolation layer to rescale the interpolated and imputed data back into the original less frequent and shorter sequence length. We also plan to explore TSI-GNN and TIBIL using appropriate missingness mechanisms as well as using other aggregation functions (e.g., LSTM, which does not necessarily assume order invariance). Further, we plan to explore combining reinforcement learning with TSI-GNN and TIBIL.

## Conclusion

Incorporating temporal information into GNN-based methods for handling missing data improved the representation, specifically when using GBR for downstream prediction tasks in regularly sampled data. We tested our method using several benchmark datasets and compared to classic and contemporary methods. We provided insight into practically implementing our proposed method by managing the size of the generated TSI-GNN. TSI-GNN outperformed GRAPE, specifically when using GBR in downstream prediction tasks in regularly sampled datasets. Our proposed method is competitive with existing temporal imputation methods.

## Data Availability

Publicly available datasets were analyzed in this study. This data can be found here: https://finance.yahoo.com/quote/GOOGL/history?p=GOOGL https://archive.ics.uci.edu/ml/datasets/Appliances+energy+prediction https://mimic.physionet.org/gettingstarted/overview/.
